# Assessment of goal-directed behavior with the 3D videogame EPELI: Psychometric features in a web-based adult sample

**DOI:** 10.1371/journal.pone.0280717

**Published:** 2023-03-21

**Authors:** Jussi Jylkkä, Liisa Ritakallio, Liya Merzon, Suvi Kangas, Matthias Kliegel, Sascha Zuber, Alexandra Hering, Juha Salmi, Matti Laine

**Affiliations:** 1 Department of Psychology, Åbo Akademi University, Turku, Finland; 2 Department of Neuroscience and Biomedical Engineering, Aalto University, Espoo, Finland; 3 Department of Psychology, University of Helsinki, Helsinki, Finland; 4 Faculty of Psychology and Educational Sciences, University of Geneva, Geneva, Switzerland; 5 Centre for the Interdisciplinary Study of Gerontology and Vulnerability, University of Geneva, Geneva, Switzerland; 6 Swiss National Center of Competences in Research LIVES—Overcoming Vulnerability: Life Course Perspectives, Lausanne & Geneva, Switzerland; 7 Department of Developmental Psychology, Tilburg University, Tilburg, The Netherlands; 8 MAGICS, Aalto University, Espoo, Finland; University of Cadiz: Universidad de Cadiz, SPAIN

## Abstract

EPELI (Executive Performance in Everyday LIving) is a recently developed gaming tool for objective assessment of goal-directed behavior and prospective memory (PM) in everyday contexts. This pre-registered study examined psychometric features of a new EPELI adult online version, modified from the original child version and further developed for self-administered web-based testing at home. A sample of 255 healthy adults completed EPELI where their task was to perform household chores instructed by a virtual character. The participants also filled out PM-related questionnaires and a diary and performed two conventional PM tasks and an intelligence test. We expected that the more “life-like” EPELI task would show stronger associations with conventional PM questionnaires and diary-based everyday PM reports than traditional PM tasks would do. This hypothesis did not receive support. Although EPELI was rated as more similar to everyday tasks, performance in it was not associated with the questionnaires and the diary. However, there were associations between time-monitoring behavior in EPELI and the traditional PM tasks. Taken together, online adult-EPELI was found to be a reliable method with high ecological face validity, but its convergent validity requires further research.

## Introduction

An important aspect of any psychological assessment method is its ability to predict real-world behaviors, that is, its ecological validity. Cognitive tasks have been criticized in this respect as they are often simplified and strictly controlled, putting emphasis on internal rather than external validity [1–4]. Moreover, traditional cognitive tasks typically use highly controlled, but therefore low-dimensional and static stimuli, which strongly stand in contrast to dynamic and complex real-world environments [5]. This reflects the common underlying assumption in traditional cognitive testing that cognitive functions are invariant across contexts, which implies that the same cognitive processes are at play in laboratory tasks and in real life [6]. This idea of generalizability is challenged by findings indicating that performances in laboratory tasks often correlate weakly with real-life behaviors, which suggests that cognitive processes may be context-sensitive [7]. This motivates a move towards a ‘cognitive ethology’ approach where cognition is tested in natural contexts [6,8]. Such studies have indeed been conducted in neuropsychology (e.g., Multiple Errands Test; [9]) but they face several practical limitations, lacking experimental control and being difficult to implement [10]. However, the development of video games and virtual reality (VR) tools has renewed interest in the development of more complex, ‘life-like’ cognitive tasks [11]. Another important aspect that has not been previously addressed in naturalistic studies is the scalability of the measurement methods. Obtaining large enough sample sizes when studying human goal-directed behavior in lifelike contexts is highly relevant: in complex task situations one can expect considerable inter-individual performance variation, and identifying factors that underlie this variation is difficult in limited samples. In addition, various background factors like age and cognitive ability, as well as the highly variable employment of spontaneous strategies could mediate the performance in complex tasks [12,13].

In the present study, we set out to examine the internal consistency as well as convergent and ecological validity of a new, Internet browser-based 3D video game called EPELI (Executive Performance in Everyday Living) in a large sample of healthy adults. A VR version of this task has been recently validated in children with ADHD versus normally developing controls [14]. However, VR methods typically require laboratory testing and significant human resources because most persons do not possess the necessary equipment. The present web-based version of EPELI enables scalable, home-based testing. Although several similar tasks have been conducted in laboratory context (for a review, see [15]), to our knowledge this is the first time when a naturalistic video game to assess cognitive processes related to goal-directed behavior is utilized remotely through the Internet.

Many of the early VR testing games were in practice implementations of laboratory tasks, such as the Wisconsin Card Sorting test [16] or the Continuous Performance Test (CPT, [17]; for a review, see [11]). However, these VR implementations suffer from many of the same limitations as traditional laboratory-based tasks, such as employing abstract, unrealistic and static stimulus materials, which lessen the task’s ecological validity. This has led to more function-led approaches which aim to simulate real-life scenarios instead of measuring some predefined psychological constructs [18]. The early attempts to develop such VR tests were based on the Multiple Errands Test (MET), a real-life test to assess executive functions [9]. In a seminal study, the Virtual Errands Test (VET; [19]) showed similar performance to MET in a small sample of participants with brain injury versus matched controls (five per group). A later study with 35 lesion patients and 35 matched controls showed that VET could reliably distinguish between the two groups [20]. Such VR tasks have then been developed and validated in numerous studies and they include, in addition to MET-type tasks, virtual shopping tasks, virtual cities, and other multitasking tests (for a review, see [11]). They have been shown to successfully detect cognitive deficits, for example, in brain damage [21], obsessive-compulsive disorder [22], or schizophrenia [23].

Like in VET, the task context in EPELI relates to prospective memory (PM), that is, remembering to perform a task in the future. This may hinge upon a specific time (time-based PM, e.g., taking medicine at 8:00am) or a certain event (event-based PM, e.g., emptying the dishwasher when it gives a signal that it has finished), or just be a list of to-do-things [24]. PM predominantly involves episodic memory in the form of remembering the intention, and executive functions (EF) such as planning the task, monitoring for cues to perform the action, and inhibiting the ongoing non-PM task to perform the intention [24–26]. PM is crucial for everyday functioning [27] and weakens in healthy aging as well as in dementia [28], and in other neurological conditions such as Parkinson’s disease [29] and autism spectrum disorder [30]. Conventional experimental tests of PM have utilized a laboratory paradigm where, as in real-life PM tasks, the participant simultaneously has to perform an ongoing task (e.g., a short-term recall task), as well as a previously instructed PM task that is either time-based (e.g. performed with one-minute intervals) or event-based (performed when a given cue appears; [31]).

Besides VET, there are also other efforts to develop ecologically valid measures of PM. For example, a recent validation study presented a virtual reality testing system called VR-EAL (Virtual Reality Everyday Assessment Lab) to assess neuropsychological performance. Performance in the VR environment was significantly associated with scores in a comprehensive paper-and-pencil test battery and VR-EAL showed ecological face validity by being rated as similar to real-life tasks [32]. In another study, researchers from the same group demonstrated the feasibility of VR-EAL to tease out the component processes in PM performance, such as delayed recognition, planning, and visuospatial attention [33]. Another noteworthy line of investigation is the Edinburgh Virtual Errands Test (EVET), a multitasking test within a simulated shopping mall and office environment. EVET loads on EF, including planning ability, and visuospatial working memory [34,35].

The present EPELI game was developed to assess PM and goal-directed behavior in an ecologically valid and scalable way. In the game, the participant is to keep in mind and perform a list of various everyday chores in a virtual apartment. In a previous VR study involving 38 children with attention deficit hyperactivity disorder (ADHD) and 38 typically developing controls, EPELI showed predictive validity, indicated by a higher rate of irrelevant actions and more controller movement in the ADHD group [14]. Moreover, EPELI showed excellent discriminatory validity with 88% area under the curve, and high correlations between EPELI performance and parent ratings of the child’s executive problems (*r* = .57) and ADHD symptoms (*r* = .55) in the whole group. Another study that tested EPELI with 77 normally developing children indicated good internal consistency and significant associations between age, gender, and verbal encoding ability [36]. Besides, worse EPELI performance was associated with parent-rated everyday executive problems also in these children, although the correlations were lower than in the previous child study [36].

### The present study

A preregistration for the present study can be found on Open Science Framework (https://osf.io/m7c9a; “Study 3”). Here we tested a new web-based version of EPELI in a sample of healthy adults. Our overall aim was to examine the reliability and validity of EPELI. Reliability was assessed by analyzing the internal consistency of the main EPELI variables (see below). Ecological validity was examined by comparing performance in EPELI to standardly used questionnaires tapping PM and EF, which were assumed to reflect the participants’ everyday PM/EF ability, as well as a diary about PM lapses that the participants kept for five days. By including the diary, we intended to alleviate problems related to retrospective questionnaires, such as failures to correctly remember PM performance in the past [37,38]. Convergent validity was tested by comparing EPELI to two conventional PM tasks, both with a time-based and an event-based version. One of the PM tasks labeled as Matching followed the paradigm introduced by Einstein and McDaniel [31], while the other called ‘Cruiser’ was game-like, based on the ‘CyberCruiser’ [39–41]. The goal of including these tasks was to examine possible differences between the traditional PM tasks, the 2-dimensional Cruiser game which hypothetically does not resemble everyday life, and the 3-dimensional EPELI game which is hypothetically more similar to everyday life. Additional tasks were two episodic memory tasks, which were included to see how strongly the PM tasks rely on episodic memory [24], and the Conners Continuous Performance Test (CPT; [42]), a widely used task to tap sustained attention. The latter task was mainly included to assess ADHD symptoms (we also tested a group of adults with ADHD that will be reported separately), but it can be hypothesized to measure in part the executive element of PM even in the control group which was at focus here. To address how much the task resembles everyday life, or its verisimilitude, we also examined the perceived lifelikeness of EPELI in comparison to the conventional laboratory PM tasks.

Our pre-registered hypothesis was that EPELI shows stronger associations with the measures of everyday PM/EF ability than conventional PM tests do. Moreover, we examined whether EPELI’s time-based and event-based PM measures are associated with the corresponding measures on the two conventional PM tasks to see whether EPELI taps on PM-related subprocesses. Additional analyses not specified in the preregistration include the predictive value of three general background factors (age, gender, and intelligence) on EPELI performance, and the verisimilitude (face validity) of EPELI as compared to conventional PM tasks.

## Method

The study was approved by the Ethics Board of the Departments of Psychology and Logopedics at the Åbo Akademi University, Finland. We recruited participants on the crowdsourcing site Prolific (prolific.co), targeting both healthy participants and adults with diagnosed ADHD (both groups of age 18–50, currently living in the UK, first language English). ADHD data will be reported elsewhere. The data gathering took place between August and December, 2021, and proceeded in three stages, two prescreens and the study proper that encompassed five sessions on five separate days.

### Prescreens and questionnaires

In the first prescreen (N = 14,443), the participants reported whether they had been diagnosed with ADHD/ADD by a clinical professional, and filled out part A of the *Adult ADHD Self-Report Scale* (ASRS; [43]). This prescreen took less than a minute and the participants were compensated with £0.17. The main purpose of the first prescreen was to identify a sufficiently large sample of adults with ADHD. Here we focus on the control participants with no ADHD. The participants were then invited to take part in the second prescreen study (N = 1,513 in the control group), where the following questions and questionnaires were administered: demographic information (age, gender, income, education); medical history (e.g., diagnosis of neurological illness, severe depression, bipolar disorder, psychosis, or schizophrenia); eyesight and color vision; alcohol use with AUDIT questions 1–3; nicotine use; and use of other psychoactive substances. Additionally, the following standardized and validated questionnaires were administered: ASRS part B to further assess ADHD problems; DSM-5 Self-Rated Level 1 Cross-Cutting Symptom Measure—Adult [44] to assess overall mental health on several dimensions; the Prospective Retrospective Memory Questionnaire (PRMQ) [45] to assess prospective and retrospective memory; and a questionnaire about the use of illicit psychoactive substances (not analyzed in the current study). The second prescreen took c. 10 minutes and the participants were compensated with £0.84.

An invitation to the experiment proper was sent only to participants who fulfilled the following eligibility criteria: normal or corrected-to-normal vision; no color blindness; no neurodevelopmental disorders; no neurological illness that affects the participant’s current life; never diagnosed with severe depression, bipolar disorder, psychosis, or schizophrenia across the lifespan; and no self-reported substance-abuse problem. Moreover, the DSM was utilized with the following eligibility criteria: no reported suicidality in DSM (i.e., score 0 in item 11), and sum scores of less than 3 in the domains depression, mania, and anxiety (i.e., at most “mild” symptoms, or a response indicating occurrence of the symptom not more than during “several days” over the last two weeks).

### The testing sessions

The actual experiment consisted of five assessment sessions. With the exception of EPELI which was always administered in the first session, and the diary questions which were administered in every session, the other tasks were fully counterbalanced between the participants by randomly allocating them into one of the four counterbalanced task sets. The five assessment sessions were performed on separate weekdays with at least a 12-hour interval in-between, and all sessions were required to be completed within 14 days. Each session took approximately 40 minutes and the complete duration of testing was c. 3 hours and 20 minutes. The participants were compensated with £16.67 for full completion of the study (partial performances were not compensated).

The first assessment session always included the EPELI game, and only those participants who completed it were allowed to partake in the other sessions. After conducting EPELI, the participants were asked about possible side-effects during the game with the Virtual Reality Sickness Questionnaire (VRSQ) [46] and their sense of presence in the game (partly based on the Presence Questionnaire; [47]).

#### EPELI game

**Fig 1:**
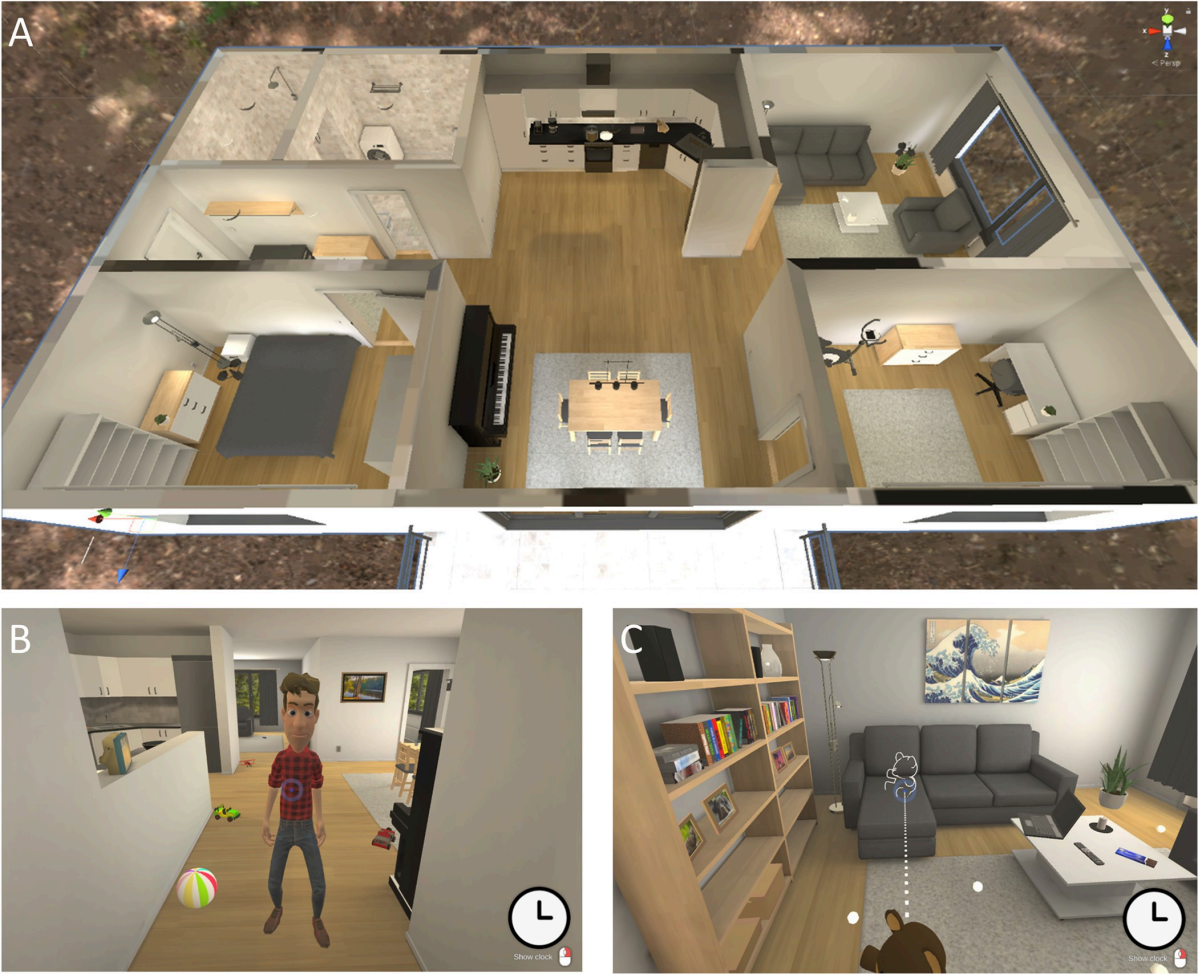
The EPELI game. In the game, the participants performed everyday chores in a virtual apartment. The floorplan (panel A) was not shown to the participant. The to-be-remembered list of tasks was given by the virtual character Vincent at the beginning of each task block (panel B). The event-based task was to place the teddy bear, if seen amidst the toys scattered on the floor, on the sofa (panel C). The participant could move around by clicking on white hotspots on the floor. A clock with running time could be opened by clicking the right mouse button.

The participants conducted the EPELI game in the first testing session after answering the diary questions and receiving technical information. They were redirected from the testing platform to the EPELI game server by clicking on a link, and the game was run in a separate browser window. The game started with guided volume adjustment, after which the participants found themselves in the lobby of a virtual apartment (see Fig 1A for the floorplan that was not shown to the participants who only had the first-person view). A virtual character, ‘Vincent’, appeared in front of the participants (see Fig 1B), welcoming them and giving instructions to the game, followed by a practice session where an apple was moved from one room to another. Visual field of view in the game was controlled by mouse and a crosshair was visible at the center of the screen. The participants moved around the apartment by clicking on white spherical hotspots on the ground, and they were able to move directly to any visible hotspot. A clock with running time (reset at the beginning of each block) was available in the lower right corner by clicking the right mouse button. Objects could be grabbed and laid on surfaces by clicking on them. Several of the objects were interactable, for example, closets that could be opened, toys scattered on the floor that could be played with, or the piano in the living room that one could play.

The EPELI task consisted of 10 blocks with eight tasks per block. In the beginning of each block, Vincent orally introduced a theme (e.g., “You have just woken up and it is time for morning activities”) and gave a list of 7–8 everyday tasks (e.g., “Brush your teeth”). Two types of PM tasks appeared in these lists: five *time-based tasks* (to be performed at a given time point) and 70 standard tasks (seven per block). The standard tasks differ from the event- and time-based tasks by not having any specified trigger event or time. Additionally, there was an ongoing event-based task which was to be performed whenever a cue was present. The cue in the event-based task was a teddy bear, which was to be put on the sofa (see Fig 1C). The teddy was present in five of the 10 blocks (never during the same block as a time-based task) and located amidst toys scattered on the floor. The instruction to this event-based task was given by Vincent only once before the game properly started. In other words, the task lists for the blocks did not include the event-based instruction anymore. Each block thus contained seven standard tasks, and additionally either one time-based task or one event-based task, making in total eight tasks in each block.

Sixty of the standard tasks were in line with the general schema of the task block that was provided by Vincent, while ten of the standard tasks (one per block) were non-schema-based, i.e., unrelated to the schema in question (e.g., during morning activities, the participants were instructed to switch off the lights in the study). This was done to reduce predictability in the task lists.

Following the earlier child version of EPELI [14], the main dependent variables (DVs) derived from EPELI were as follows: 1) total score (correctly performed subtasks), 2) task efficacy (percentage of relevant actions out of all actions excluding clicks on the waypoints that enable moving around in the environment; ‘relevant’ means actions that contribute to completing the given tasks), 3) navigation efficacy (total score divided by covered distance), 4) mouse motion, 5) actions (total number of both task-relevant and -irrelevant clicks), 6) time-based subtask score, 7) number of clock checks for the blocks with a time-based subtask, and 8) event-based subtask score. After each block, the participants answered an open question as to how they performed the task. These responses were used to identify strategy employment in the blocks, and these data will be reported elsewhere.

After the EPELI session, the other four assessment sessions contained tasks and questionnaires tapping on, e.g., EF and PM, as listed below.

#### Conventional PM measures (Cruiser and Matching)

The overall structure of these two tasks was as follows. At first, there was a practice round of the ongoing task (or ‘main task’), after which instructions for the actual PM task (i.e., the task that must be conducted in parallel with the ongoing task) were given (e.g., “next time you perform this task, you must also remember to refuel the car when you see yellow flowers”). The PM instructions were provided only once. Following the instructions, an intermediate task (a questionnaire) was administered (this is called the *delay phase*), the purpose of which is to flush working memory and to ensure that the PM instruction was stored in long-term (episodic) memory. After this, the actual PM task was presented, where the ongoing task was coupled with the to-be-remembered PM task (e.g., to refuel). At the end of each PM task, the participants were asked to repeat the task instructions. This was done to ensure that the instructions had been stored in long-term memory, and that a possible failure to conduct the PM task was due to actual PM failure instead of a simple long-term memory failure.

*Cruiser*. The structure of this task resembles conventional lab-based PM tasks [31], albeit it was designed as a simple video game. In this task, the participants drove a car on a multilane road in busy traffic, overtaking other cars (scoring points) and trying to avoid hitting them (losing points). The task was based on the CyberCruiser [39], with task structure mainly based on the Dresden Cruiser version [40,41]. In the *time-based version*, the PM task was to refuel when the gauge showed that the fuel tank was getting close to being empty (indicated by the gauge turning red). This happened at 60-second intervals and for 5 cycles, making the total task length 5 minutes. In the *event-based version*, the participants were instructed to refuel when a bed of yellow flowers was seen on either side of the street. A target (yellow flowers) appeared at five pseudorandom times during the five-minute duration of the whole task. The main DV was the accuracy, which was defined as the number of correct PM responses, calculated separately for each of the two task variants. Additionally, the time-based version yielded time-monitoring behavior (i.e., the number of times that the gauge was checked).

*Matching*. This task resembles conventional lab-based PM tasks [31] not only in structure but also by its layout. Like the Cruiser, it includes a time-based and an event-based version. Also similarly to the Cruiser, there is a PM task that is to be performed in parallel with an ongoing primary task. Here the primary task requires the participants to determine if two simultaneously shown colored symbol strings (e.g., "874G2" and "834G2", displayed with different font colors) are identical or not (irrespective of color). In the time-based variant, the PM task should be performed whenever a timer shows the last 5 seconds of a given minute. The total task duration was 5 minutes and it included five PM targets. As in the Cruiser, the timer is briefly visible by pressing a specific button. In the *event-based* variant, the target button should be pressed each time one of the symbol strings is red and the other one is blue (this happens at five random times during 5 minutes). The main DV was accuracy defined as the number of correct PM responses, calculated separately for the time-based and the event-based version (however, note that some PM responses were excluded based on criteria defined below). Furthermore, the time-based version yielded a measure of time-monitoring (i.e., the number of times that the timer was checked).

#### Word list learning task (WLL)

This task taps on episodic memory [48]. The participants were presented with a list of 18 words that they must recall after an intermediate arithmetic task. Each word was presented for one second, with an inter-stimulus-interval of one second. There were three blocks with the same words presented in random order. The main dependent variable was the average number of words recalled. After each block, the participant reported the strategies they used in the task (not reported here).

#### Instruction recall task (IR)

This task was used to measure the episodic memory component in the EPELI game, separately from “memory in action” that characterizes EPELI performance [14]. Here the participant is to recall a list of eight EPELI-like instructions immediately after they have been presented, one at a time on the screen. The main dependent variable was the number of correctly recalled instructions written down by the participants.

#### The International Cognitive Ability Resource with 16 items (ICAR16)

This test [49] was used to measure participants’ general cognitive ability.

**Conners Continuous Performance Test (CPT)** with letters [50]. This is a commonly used test of sustained attention especially in the context of ADHD.

#### Ratings of the performance tasks

After each task, the participants rated its verisimilitude, i.e., likeness to real life (“How much did the task resemble your everyday life?”, answered on a seven-point scale). In addition, they assessed task difficulty and motivation (answered on five-point scales).

#### Questionnaires

In the questionnaires, the participants assessed their everyday cognitive functioning concerning PM, executive functions, metacognition, anxiety and depressive symptoms. The questionnaires included in the five-day testing sessions were the following:

*Adult Executive Functioning Inventory* (ADEXI) [51] yields two subscales, working memory and inhibition.

*Metacognitive Prospective Memory Inventory* (shortened version; MPMI-s) [52] yields three subscales: ability (PMA), use of internal aids (PMSi), and use of external aids (PMSe).

*International Personality Item Pool* (IPIP), subscale for the anxiety construct [53].

*The 16-Item Quick Inventory of Depressive Symptomatology* (QIDS-SR16) to assess depressive symptoms [54].

#### Diary questions about everyday PM

In the beginning of each of the five testing sessions, the participants reported their prospective memory errors during the last 24 hours and responded to a question probing how stressful their day was. We used six questions, constructed on the basis of the findings by [37]. The questions were the following: 1) Forgot an appointment or other action scheduled for a specific time (e.g., medical appointment, forgot to turn on the TV for one’s favorite show); 2) Forgot to do something involving people that was not scheduled for a specific time (e.g., forgot to call a friend or send an email); 3) Forgot to do something not involving people that was not scheduled for a specific time (e.g., to put the laundry in the washing machine, post a letter); 4) Forgot what one was doing (e.g., went to the kitchen and forgot what one came to get); 5) Repeatedly carried out an already completed action (e.g., took medication twice or asked the same question twice in a row); 6) Almost forgot to carry out something (e.g., almost forgot to print out important documents for a meeting). The response options were as follows: zero times during the last 24 hours; 1–2 times during the last 24 hours; 3–4 times during the last 24 hours; 5–6 times during the last 24 hours; or 6+ times during the last 24 hours. These questions were employed to probe the participants’ everyday PM lapses, using the five testing days as the sampling period.

### Analytical approach

In accordance with the preregistration, Bayes factors (BF) instead of frequentist statistics were calculated for each analysis. BF_10_ indicates the likelihood of the observed data if the alternative hypothesis holds, in proportion to its likelihood if the null hypothesis is true. BF_10_ is mathematically defined as follows:

$$BF_{10}=P(D|H_{1})/P(D|H_{0})$$

where P is likelihood, D is data, and H1 and H0 are the alternative and null hypotheses, respectively. For example, if BF_10_ = 3, the data is three times more likely if the alternative hypothesis is true. Conversely, the inverted BF, i.e., BF_01_, indicates the likelihood of the data if the null hypothesis is true compared to if the alternative hypothesis is true. The BF_10_ is interpreted as follows: > 100 Extreme evidence for H_1_; 30–100 Very strong evidence for H_1_; 10–30 Strong evidence for H_1_; 3–10 Moderate evidence for H_1_; 1–3 Anecdotal evidence for H_1_; 1 No evidence. The inverted BF is interpreted in the same way, but the evidence is for H_0_ [55].

### Sample and exclusions

The sample size before further exclusions was 293, of which 246 had full and 47 had incomplete data. All the incomplete participant data included at least the first session (EPELI). We removed all participants who reported that they had cheated or were intoxicated in any of the sessions. This resulted in deleting 32 participants (11%), resulting in *N* = 261. Additionally, six participants were removed due to missing background data. Thus, the final *N* was 255, of which 210 had performed all the sessions and the rest had performed at least the EPELI session.

In the cognitive tests (but not in the questionnaires), we removed as univariate outliers all observations that were three standard deviations (3 SD) away from the group mean. Additionally, in the PM tasks (Cruiser and Matching), the prospective memory task performance (i.e., pressing the target button or refueling) was defined as an outlier if the participant had an extreme (i.e., ≥ 3 SD deviance from the group mean) amount of false alarms (i.e., performances of the PM task outside the target time or when the cue was not present), or if their performance in the main task was an extreme outlier (i.e., ≥ 3 SD deviation from the group mean in the number of crashes in Cruiser or in the rate of correct responses in Matching), or if they failed to correctly recall what they were supposed to do in the task. These criteria resulted in the deletion of 14/1895 observations (0.73%) across all eight EPELI variables, and 8/633 observations (1.3%) over the three CPT variables. As to the Cruiser tasks, we removed 57/212 observations (27%) in the Cruiser event-based PM variable (of which 56 were due to failure to recall instructions), 17/212 observations (8.0%) in the Cruiser time variable “refills” (of which 14 due to failure to recall instructions), and 3/212 observations (1.4%) in the “fuel checks”. In the Matching task versions, we removed 45/214 observations (21%) in Matching event-based variant (of which 43 due to failure to recall instructions), and 63/214 observations (29%) in the Matching time-based variant (of which 58 due to failure to recall instructions). Note that in the Matching time-based variant, time monitoring behavior could not be examined because that data was not saved due to a programming error. Finally, 3/633 observations (0.47%) were removed in the Word List Learning task and none were removed in the Instruction Recall task.

## Results

The final sample consisted of 255 participants (185 females, 73%). Their average age was 31.80 years (*SD* = 8.71) and they had on average 16.37 years of education (*SD* = 3.05). More precisely, 46% reported that they had a bachelor’s degree; 18% had a master’s degree; 17% higher secondary (e.g., high school diploma); 10% reported vocational university or other upper vocational education; frequencies in the rest of the categories were < 5%. Their self-estimated income level was slightly below average (*M* = 2.57; *SD* = .99 on a scale from 1 to 5; 1 = Much below average, 2 = Below average, 3 = Average, 4 = Above average, 5 = Much above average).

Average scores in the questionnaires are summarized in Table 1 and average performance in the EPELI game is summarized in Table 2. Performance in the other PM tasks is summarized in Table 3, and performance in CPT and the episodic memory tasks in Table 4.

**Table 1:** Average responses to the questionnaires that probe into executive functions and prospective memory.

	ASRS A	PRMQ-P	PRMQ-R	ADEXI Total	ADEXI-I	ADEXI-WM	MPMI-PMA	MPMI-PMSE	MPMI-MPSI	Diary: PM lapses
N	255	255	255	214	214	214	213	213	213	208
Missing	0	0	0	41	41	41	42	42	42	47
Mean	1.682	2.438	2.132	2.523	2.486	2.544	2.129	2.394	2.602	7.586
St.dev.	0.642	0.723	0.67	0.521	0.558	0.608	0.57	0.718	0.745	1.729
Min	0.167	1	1	1.286	1	1.222	1	1	1	6
Max	4	5	4.75	4.714	4.6	4.778	3.625	4.571	4.714	21.4

Note: ASRS = Adult ADHD Self-Report Scale Part A (range from 0 to 4); PRMQ = The Prospective Retrospective Memory Questionnaire (P = prospective subscale; R = retrospective subscale) (range from 1 to 5); ADEXI = Adult Executive Functioning Inventory (Total = Total score, I = Inhibition subscale; WM = Working Memory subscale) (range from 1 to 5); MPMI = Metacognitive Prospective Memory Inventory (PMA = prospective memory ability; PMSE = use of external aids; PMSI = use of internal aids;) (range from 1 to 5). The mean value in the diary reflects the average amount of PM lapses made per day during the five days (theoretical range from 6 to 30 where 6 reflects no PM lapses; see the subsection *Diary questions about everyday PM* for details).

**Table 2:** Performance in EPELI.

	Total score	Task efficacy	Navigation efficacy	Controller motion	Total actions	Correct event-based tasks	Correct time-based tasks	Looked at the watch
N	244	243	243	242	237	244	243	242
Missing	11	12	12	13	18	11	12	13
Mean	55.619	0.369	0.051	48976.732	484.316	2.93	4.23	21.682
St.dev.	11.341	0.112	0.012	12709.65	123.894	2.03	0.785	11.321
Min	26	0.086	0.02	21143.29	209	0	2	1
Max	79	0.682	0.083	85401.88	914	5	5	57

**Table 3:** Performance on the other PM tasks.

	**Cruiser**								
	**Event**				**Time**				
	**Hits**	**False alarms**	**Recalled instruction?**	**Main task collisions**	**Hits**	**False alarms**	**Recalled instruction?**	**Main task collisions**	**Check fuel**
N	155	208	213	211	195	211	213	210	209
Missing	100	47	42	44	60	44	42	45	46
M/%	3.497	21.875	74%	36.621	3.446	3.427	94%	36.919	26.292
SD	1.336	29.609	-	11.969	1.52	6.687	-	11.301	12.589
Min	0	0	-	12	0	0	-	14	0
Max	5	161	-	75	5	51	-	69	71
	**Matching**								
	**Event**				**Time**				
	**Hits**	**False alarms**	**Recalled instruction?**	**Main task correct**	**Hits**	**False alarms**	**Recalled instruction?**	**Main task correct**	**Check time**
N	169	209	212	207	151	210	214	210	-
Missing	86	46	43	48	104	45	41	45	-
M/%	3.402	2.383	81%	36.087	2.828	1.643	73%	69.538	-
SD	1.342	4.676	-	3.293	1.33	2.242	-	7.353	-
Min	0	0	-	20	0	0	-	39	-
Max	5	22	-	40	4	14	-	79	-

Note: In the main task “hits” variable, we have excluded the following: Those who are extreme outliers on 1) the named variable or 2) on performance in the main task, or 3) false alarms, or 4) who have failed to remember the PM task. Theoretical ranges in each PM variable are from 0 to 5. “Recalled instruction?” refers to whether the participant correctly remembered the PM task instruction. The sample size is lower in the presented tasks compared to EPELI, due to dropout after the EPELI session which was always presented as first.

**Table 4:** Performance in CPT and the episodic memory tasks.

	CPT commissions	CPT Mean SD	CPT omissions	IR correct	WLL correct
N	210	207	208	217	211
Missing	45	48	47	38	44
Mean	15.557	97.94	4.591	4.06	11.742
St.dev.	6.242	43.477	8.274	1.605	2.7
Min	0	44.175	0	0	3
Max	33	302.394	69	8	17.333

*Note*: CPT = Conners Continuous Performance Test; IR = Instruction Recall (number of correctly recalled items); WLL = Word List Learning (average number of correctly recalled words). The sample size is lower in CPT compared to EPELI, due to dropout after the EPELI session which was always presented as first.

Variable distributions were examined visually with histograms. There was some evidence of floor effect with PRMQ. In the PM diary there was a clear floor effect. Based on the Shapiro-Wilk test, of all the questionnaires and PM diary, only the Prospective Memory Strategies Internal (PMSi) subscale of the MPMI was normally distributed. The EPELI variables did not show clear floor- or ceiling effects, except for Correct time-based tasks, which had a slight ceiling effect. Moreover, the Correct event-based tasks variable was bimodal. Out of the EPELI variables, only the efficacy measures were normally distributed based on the Shapiro-Wilk test. As to the conventional PM tasks, the dependent variables in each task appeared to be limited by a ceiling effect. We then examined if this was due to the strict exclusion criteria by looking at the same variables with looser exclusion criteria (e.g., where we did not exclude the participants who had failed to remember the PM task instruction), but this only increased error variance and did not remove the ceiling effect. None of the conventional PM task variables were normally distributed. Of the CPT variables, only the Commissions variable was normally distributed. Omissions variable, in turn, showed a floor effect. As Bayesian Pearson correlations do not assume normality and those are more sensitive than non-parametric tests, we nevertheless used this method to examine associations between the variables. Non-parametric Kendall’s taus were also examined for comparison, given the non-normality of most variables. The results remained largely the same, albeit the estimated correlations were smaller by .1 on average.

We then examined how similar the participants considered the PM tasks to be with their everyday life on a scale from 1 to 7, as well as the perceived difficulty and motivation in the tasks on a scale from 1 to 5, summarized in Table 5. EPELI was rated as more similar to everyday life than the other PM tasks, with decisive Bayesian evidence (BF_10_s > 100).

**Table 5:** Self-rated difficulty, familiarity, and motivation in the tasks.

	Difficulty	Familiarity	Motivation
	**EPELI**		
N	255	255	255
Mean (SD)	3.557 (0.945)	4.063 (1.215)	4.063 (0.89)
	**CPT**		
N	211	211	211
Mean (SD)	3.872 (1.018)	1.479 (0.922)	3.64 (1.144)
	**Cruiser event**		
N	213	213	213
Mean (SD)	2.869 (1.047)	2.056 (1.152)	4.329 (0.827)
	**Cruiser Time**		
N	214	214	214
Mean (SD)	2.897 (0.997)	2.266 (1.373)	4.341 (0.856)
	**Matching Event**		
N	213	213	213
Mean (SD)	3.474 (0.998)	1.718 (0.998)	4.141 (0.905)
	**Matching Time**		
N	214	214	214
Mean (SD)	3.308 (1.121)	1.822 (1.028)	4.164 (0.897)
	**Instruction Recall**		
N	218	218	218
Mean (SD)	3.706 (0.929)	4.206 (1.436)	4.161 (0.83)
	**Word List Learning**		
N	212	212	212
Mean (SD)	3.656 (0.826)		2.783 (1.421)		4.552 (0.647)

*Note*: Similarity to everyday life theoretical range from 1 to 7; in the other variables from 1 to 5. The sample size is lower in the other tasks besides EPELI, due to dropout after the EPELI session which was always presented as first.

In VRSQ, the participants did not report any substantial virtual reality sickness in EPELI, having an average score of .36 (SD = .40) on a scale from 0 (“not at all”) to 3 (“very much”). Their average score in the Presence Questionnaire was 4.96 (SD = .85) on a scale from 1 (not at all) to 7 (completely).

### Internal reliability and intervariable correlations in EPELI

As estimated by Cronbach’s alpha coefficients, internal reliability of the EPELI variables was high over the ten task blocks: Total Score α = .878; Task efficacy α = .807; Navigation Efficacy α = .807; Controller Motion α = .956; Total Actions α = .947. Alphas for time- and event-based tasks were not assessed, because their value is 1/0 and they occur only in 5 of the blocks; also alpha was not calculated for Time Monitoring, which only applies to blocks with time-based tasks (i.e., 5 blocks). Considering future needs for clinically more viable shortened versions of EPELI, reliabilities as a function of number of blocks are reported in S1 Table. Associations between all of the EPELI variables (over all the blocks) are summarized in Table 6. Of notice is that Task efficacy and Navigation efficacy correlated very strongly (*r* > .9), indicating that they measure the same construct. As expected, total number of actions and controller motion correlated negatively with the task efficacy measures.

**Table 6:** Associations between the EPELI variables.

Variable		Total Score		Task Efficacy		Navigation Efficacy		Controller Motion		Total Actions		Correct Event-based tasks		Correct Time-based tasks	
Total Score	Pearson’s r	—													
	BF₁₀	—													
Task Efficacy	Pearson’s r	0.485	***	—											
	BF₁₀	6.26E+12		—											
Navigation Efficacy	Pearson’s r	0.453	***	0.905	***	—									
	BF₁₀	6.85E+10		4.88E+87		—									
Controller Motion	Pearson’s r	0.372	***	-0.29	***	-0.423	***	—							
	BF₁₀	3.99E+06		2759.063		1.09E+09		—							
Total Actions	Pearson’s r	0.374	***	-0.457	***	-0.507	***	0.807	***	—					
	BF₁₀	3.51E+06		5.11E+10		7.70E+13		1.32E+52		—					
Correct Event-based tasks	Pearson’s r	0.57	***	0.238	**	0.177		0.316	***	0.276	***	—			
	BF₁₀	2.60E+19		83.047		3.588		22202.203		807.3		—			
Correct Time-based tasks	Pearson’s r	0.415	***	0.066		0.05		0.277	***	0.275	***	0.227	**	—	
	BF₁₀	5.38E+08		0.135		0.108		1063.816		725.637		43.927		—	
Looked at the watch (times)	Pearson’s r	0.378	***	-0.102		-0.098		0.448	***	0.463	***	0.234	**	0.227	**
	BF₁₀	7.33E+06		0.28		0.256		2.44E+10		1.04E+11		64.59		42.812	

* BF₁₀ > 10

** BF₁₀ > 30

*** BF₁₀ > 100.

### Testing the hypothesis of EPELI’s stronger associations with self-assessed everyday PM

We examined the ecological validity of EPELI by comparing it to the questionnaires and the PM diary data. There was no evidence for associations (all BF_10_’s < 3). Instead, in almost all variable pairs there was moderate (BF_01_ > 3) to strong (BF_01_ > 10) evidence for the *absence* of an association. Correlation table with inverted Bayes Factor is presented in Table 7.

**Table 7:** Associations between the EPELI variables and the questionnaires and the PM diary. The Bayes Factors reported here are inverted (BF_01_) and indicate evidence for the null hypothesis.

Variable		Total Score	Task Efficacy	Navigation Efficacy	Controller Motion	Total Actions	Correct Event-based tasks	Correct Time-based tasks	Looked at the watch (times)
ASRS A	Pearson’s r	0.057	0.003	0.034	4.60E-04	0.032	-1.01E-04	-0.023	0.037
	BF₀₁	8.447	12.441	10.877	12.425	10.873	12.476	11.713	10.546
PRMQ Prospective	Pearson’s r	-0.069	-0.036	-0.014	-0.061	-0.077	-0.025	0.033	0.012
	BF₀₁	7.071	10.67	12.161	7.968	6.129	11.555	10.917	12.205
PRMQ Retrospective	Pearson’s r	-0.057	-0.132	-0.108	0.048	0.063	0.015	0.005	0.041
	BF₀₁	8.473	1.52	3.045	9.478	7.759	12.146	12.412	10.145
ADEXI Total	Pearson’s r	-0.075	-0.084	-0.109	-0.004	0.007	-0.039	0.125	-0.075
	BF₀₁	6.437	5.593	3.429	11.45	11.295	9.874	2.326	6.458
ADEXI Inhibition	Pearson’s r	-0.005	-0.014	-0.053	0.048	0.035	-0.024	0.096	-0.022
	BF₀₁	11.489	11.263	8.627	9.11	10.024	10.849	4.496	10.904
ADEXI WM	Pearson’s r	-0.097	-0.104	-0.117	-0.03	-0.009	-0.039	0.116	-0.087
	BF₀₁	4.364	3.794	2.835	10.482	11.269	9.857	2.872	5.316
MPMI PMA	Pearson’s r	-0.102	-0.082	-0.089	-0.005	-0.021	-0.026	0.004	0.009
	BF₀₁	3.955	5.845	5.177	11.407	10.844	10.741	11.447	11.348
MPMI PMSE	Pearson’s r	0.097	-0.051	-0.115	0.161	0.128	0.024	0.06	0.038
	BF₀₁	4.403	8.773	3.008	0.828	2.247	10.862	7.996	9.894
MPMI PMSI	Pearson’s r	0.083	-0.08	-0.117	0.182	0.1	0.029	0.059	0.075
	BF₀₁	5.692	6.033	2.849	0.385	4.223	10.532	8.084	6.479
Diary average (PM lapses)	Pearson’s r	-0.122	-0.087	-0.045	-0.032	-0.059	-0.092	-0.109	0.004
	BF₀₁	2.593	5.332	9.239	10.186	7.985	4.921	3.453	11.276

Note: ASRS A = Adult ADHD Self-Report Scale Part A (range from 0 to 4); PRMQ = The Prospective Retrospective Memory Questionnaire (Prospective memory subscale; Retrospective memory subscale) (range from 1 to 5); ADEXI = Adult Executive Functioning Inventory (Total score, Inhibition = Inhibition subscale score, WM = Working Memory subscale score) (range from 1 to 5); MPMI = Metacognitive Prospective Memory Inventory (PMA = prospective memory ability; PMSE = use of external aids; PMSI = use of internal aids) (range from 1 to 5). Diary average (PM lapses) represents the average amount of PM lapses made per day during the five days (theoretical range from 6 to 30 where 6 reflects no PM lapses; see the subsection Diary questions about everyday PM for details).

Since we expected EPELI to be more strongly associated with measures of everyday PM than the traditional PM tasks, we also analyzed the correlations between the traditional PM tasks and the questionnaires and diary, summarized in Table 8. There was no evidence for associations, but instead moderate (BF_01_ > 3) to strong (BF_01_ > 10) evidence for the lack of association.

**Table 8:** Associations between the conventional PM tasks and CPT with the questionnaires. Inverted Bayes Factors (BF_01_) are reported, indicating evidence for the null hypothesis.

Variable		CT Refills	CT Fuel checks	CE Refills	MT PM hits	ME PM hits	CPT Commission errors	CPT Mean STD	CPT Omission errors
ASRS	Pearson’s r	-0.103	0	-0.021	0.017	-0.001	0.08	0.029	-0.058
	BF_01_	4.049	11.494	9.615	9.615	10.417	5.952	10.526	8.197
PRMQ Prospective	Pearson’s r	-0.064	0.034	-0.004	-0.017	-0.098	-0.019	-0.013	-0.088
	BF_01_	7.576	10.309	9.901	9.615	4.717	11.111	11.364	5.263
PRMQ Retrospective	Pearson’s r	-0.051	0.027	0.008	0.074	-0.086	0.004	0.053	-0.038
	BF_01_	8.696	10.753	9.901	6.536	5.65	11.494	8.621	9.901
ADEXI Total	Pearson’s r	-0.024	0.058	-0.003	-0.039	-0.058	-0.001	0.042	-0.14
	BF_01_	10.526	8.197	9.901	8.772	7.874	11.494	9.524	1.555
ADEXI Inhibition	Pearson’s r	0.016	0.068	-0.008	0.046	0.008	0.006	0.103	-0.12
	BF_01_	10.87	7.194	9.804	8.403	10.309	11.494	3.906	2.646
ADEXI WM	Pearson’s r	-0.04	0.042	0	-0.071	-0.082	-0.005	0.005	-0.125
	BF_01_	9.524	9.615	9.901	6.757	5.917	11.494	11.494	2.309
MPMI PMA	Pearson’s r	-0.075	-0.05	0.013	0.029	-0.099	-0.063	0.013	-0.119
	BF_01_	6.494	8.929	9.804	9.174	4.651	7.692	11.364	2.695
MPMI PMSE	Pearson’s r	0.089	-0.064	-0.008	0.131	0.038	0.09	-0.016	-0.077
	BF_01_	5.181	7.576	9.901	2.755	9.174	5.025	11.236	6.289
MPME PMSI	Pearson’s r	0.148	0.043	-0.048	0.126	0.049	0.093	-0.039	-0.136
	BF_01_	1.362	9.524	8.333	3.086	8.475	4.762	9.901	1.733
Diary Average	Pearson’s r	-0.113	-0.024	0.091	-0.066	-0.078	-0.069	0.032	-0.039
	BF_01_	3.367	10.753	5.319	7.092	6.289	7.092	10.309	9.804

Note: ASRS A = Adult ADHD Self-Report Scale Part A (range from 0 to 4); PRMQ = The Prospective Retrospective Memory Questionnaire (Prospective memory subscale; Retrospective memory subscale) (range from 1 to 5); ADEXI = Adult Executive Functioning Inventory (Total score, Inhibition = Inhibition subscale score, WM = Working Memory subscale score) (range from 1 to 5); MPMI = Metacognitive Prospective Memory Inventory (PMA = prospective memory ability; PMSE = use of external aids; PMSI = use of internal aids) (range from 1 to 5). Diary average (PM lapses) represents the average amount of PM lapses made per day during the five days (theoretical range from 6 to 30 where 6 reflects no PM lapses; see the subsection Diary questions about everyday PM for details).

To examine possible associations between depressive symptoms and self-reported PM/EF problems, we did unplanned correlation analyses on the depression scores (measured with the Quick Inventory of Depressive Symptomatology; QIDS) and the self-reports of PM/EF. Except for MPMI, there was definite evidence (BF_10_s > 100) for a positive association between all the PM/EF self-reports and the QIDS depression scores, ranging from *r* = .27 (ADEX inhibition) to *r* = .43 (ASRS B-section average). To check whether this collinearity could account for the lack of associations between the PM/EF questionnaires and EPELI, we examined in multiple regression models whether EPELI total score was associated with any of the PM/EF questionnaires when the QIDS score was also included as predictor. None of the models significantly predicted variance in the EPELI total score (BF_01_s > 7).

### Relationships between EPELI variables and the other cognitive tasks

We next examined the research question of whether the time- and event-based variables in EPELI are associated with the respective variables in the traditional PM tasks, as well as EPELI’s relationships with the episodic memory task and CPT.

All inter-task correlations are summarized in Table 9. Correct time-based tasks in EPELI were not associated with Cruiser Time refills (BF_10_ = .65; BF_01_ = 1.54) nor with Matching Time PM hits (BF_10_ = .10; BF_01_ = 9.62), but time monitoring in EPELI (Looked at watch) was associated with Cruiser Time fuel checks (r = .22, BF_10_ = 10.19), and Matching time PM hits (r = .27, BF_10_ = 41.44). Event-based task performance in EPELI was not associated with Cruiser event refills (BF_10_ = .10, BF_01_ = 9.80) or with Matching event PM hits (BF_10_ = .12, BF_01_ = 8.20).

**Table 9:** All pairwise correlations between EPELI and the performance tasks.

Variable		Total Score		Task Efficacy		Navigation Efficacy		Controller Motion		Total Actions		Correct Event-based tasks	Correct Time-based tasks	Looked at the watch (times)	
**CE Refills**	Pearson’s r	0.103		0.072		0.098		-0.062		-0.03		-8.36E-04	0.076	0.093	
	BF₁₀	0.223		0.15		0.206		0.135		0.11		0.102	0.156	0.191	
**CT Refills**	Pearson’s r	0.262	**	0.187		0.146		0.171		0.006		0.131	0.145	0.108	
	BF₁₀	66.825		2.42		0.657		1.388		0.093		0.451	0.653	0.271	
**CT Fuel Checks**	Pearson’s r	0.269	***	0.169		0.114		0.137		0.073		0.106	0.089	0.217	*
	BF₁₀	153.807		1.565		0.323		0.569		0.15		0.27	0.192	10.185	
**ME PM Hits**	Pearson’s r	0.066		0.064		0.086		-0.035		-0.007		0.052	0.023	0.112	
	BF₁₀	0.139		0.136		0.177		0.108		0.099		0.122	0.102	0.267	
**MT PM Hits**	Pearson’s r	0.199		-0.029		-0.109		0.279	**	0.3	**	0.128	0.01	0.284	**
	BF₁₀	1.847		0.11		0.241		31.821		69.511		0.339	0.104	41.443	
**CPT Commission errors**	Pearson’s r	0.118		-0.088		-0.046		-0.084		0.119		0.197	0.071	0.088	
	BF₁₀	0.355		0.19		0.108		0.178		0.352		4.6	0.145	0.189	
**CPT Mean STD**	Pearson’s r	-0.233	*	-0.255	**	-0.165		-0.075		0.002		-0.078	0.05	-0.132	
	BF₁₀	21.889		65.473		1.305		0.155		0.09		0.161	0.113	0.497	
**CPT Omission errors**	Pearson’s r	0.003		-0.126		-0.094		0.032		0.084		0.006	0.016	-0.003	
	BF₁₀	0.088		0.423		0.21		0.098		0.175		0.088	0.091	0.089	
**IR Correct**	Pearson’s r	0.265	***	0.284	***	0.271	***	4.90E-04		-0.057		0.087	0.052	0.02	
	BF₁₀	162.287		506.095		218.607		0.087		0.121		0.189	0.115	0.09	
**WLL Correct**	Pearson’s r	0.263	***	0.311	***	0.307	***	0.016		-0.115		0.181	-0.016	-0.045	
	BF₁₀	116.102		2254.069		1762.887		0.09		0.322		2.499	0.09	0.108	

* BF₁₀ > 10

** BF₁₀ > 30

*** BF₁₀ > 100.

Note: CE = Cruiser Event-based prospective memory; CT = Cruiser Time-based prospective memory; ME = Matching Event-based prospective memory; MT = Matching Time-based prospective memory; CPT = Continuous Performance Test; IR = Instruction Recall (number of correctly recalled items); WLL = Word List Learning (average number of correctly recalled words).

Of the three CPT variables, only the variance measure (Mean STD) was correlated with two EPELI measures. CPT Mean STD correlated negatively with EPELI Total Score (r = -.23, BF_10_ = 22) and Task Efficacy (r = -.26, BF_10_ = 65). The other CPT variables did not show evidence for associations with EPELI.

The episodic memory tasks showed relatively consistent correlations with EPELI Total Score as well as Task- and Navigation Efficacy. Instruction Recall correlated positively with EPELI Total Score, Task efficacy, and Navigation Efficacy (r’s > .26, BF_10_’s > 160). Word List Learning correlated also with the same EPELI variables (r’s > .26, BF_10_’s > 116).

### Relationships between background factors and EPELI performance

We also performed some additional analyses not listed in the pre-registration. To explore general determinants of EPELI performance, we ran multiple regression analyses to see if the background characteristics age, gender, and general intelligence (i.e., ICAR score) predict EPELI scores. Five separate multiple regression models were run for the five EPELI variables Total Score, Task Efficacy, Navigation Efficacy, Controller Motion, and Total Actions (see Table 10). The models significantly predicted variance in all EPELI variables. Higher age was associated with lower Total Score, as well as less Controller Motion and Total Actions. Female gender predicted better Task- and Navigation Efficiency, as well as less Controller Motion and Total Actions. Higher general intelligence predicted better Total Score, Task Efficacy, and Navigation Efficacy, but was not associated with Controller Motion or Total Actions (the *p*-value indicated that higher intelligence would predict less Total Actions, but this was not supported by the Bayes Factor).

**Table 10:** Associations between the background factors age, gender, and intelligence (ICAR score) and the key EPELI variables.

	Total score		Task efficacy		Navigation efficacy		Controller motion		Total actions	
	Beta	*BF10*	Beta	*BF10*	Beta	*BF10*	Beta	*BF10*	Beta	*BF10*
Age	-.27***	>100	.062	.35	.062	.33	-.27***	>100	-.42***	>100
Gender (1 = female)	-.13	1.09	.15*	2.23	.27***	>100	-.40***	>100	-.35***	>100
ICAR total score	.21**	22.13	.26***	>100	.26***	>100	-.060	.26	-.14*	1.97
*F*	12.45***	>100	5.73***	18.23	8.90***	>100	21.36***	>100	30.16***	>100

*Note a*:

* p < .05

** p < .01

*** p < .001.

*Note b*: In calculating BF for individual predictors, each predictor is compared to a null model that includes all the other predictors. Full model BF is calculated by comparing the model with all predictors to the null model.

*Note c*: One participant who reported “other” as gender has been excluded.

### Associations between the self-report measures of PM

Finally, we examined to what extent the self-reports of PM ability were correlated, as there were concerns on floor effects some in the PM diary and PRMQ. All the questionnaires and the diary reports were positively correlated (r’s > .29, BF_10_’s > 100), except for the strategy measures of MPMI (i.e., PMSi and PMSe), which did not show associations. All correlations are reported in S2 Table.

## Discussion

The aim of the present study was to examine the internal consistency as well as ecological and convergent validity of the EPELI game that was designed to measure EF and goal-directed behavior, as well as PM. The creation of this task was motivated by the lack of ecological validity of traditional EF and PM tasks, and the need for scalable measurement instruments that are more similar in structure and stimuli to everyday tasks. Our hypothesis, based on the results from the previous child version of EPELI that was administered with VR goggles [14,36], was that adult EPELI would show stronger associations with everyday EF and PM performance, measured with questionnaires and the diary, than the traditional PM tasks would do. The diary was included to provide a more direct measure of everyday PM performance, given the apparent difficulty of remembering memory failures in questionnaires that probe a long time span in the past. Additionally, we aimed to examine whether the time- and event-based variables in EPELI are associated with the respective traditional PM tasks, and to compare EPELI to CPT and two episodic memory tasks. As a post hoc analysis, we examined whether EPELI performance was predicted by the general background factors age, gender, and intelligence.

The results can be summarized as follows. (1) EPELI showed good to excellent *internal consistency*. As shown in S1 Table, even shorter versions of EPELI provide reasonable reliability. (2) With regard to ecological validity, EPELI was rated as two times more similar to everyday life than the classical PM tasks, giving support to its verisimilitude. However, EPELI did not show evidence for associations with the EF and PM questionnaires or the diary that were assumed to tap on everyday EF and PM performance. Neither were the traditional PM task performances or CPT associated with the questionnaires or PM diary. Instead, there was moderate to substantial evidence for lack of associations, which led us to reject our hypothesis. Possible reasons for this finding are discussed below. (3) Analysis of EPELIs correlations with the other cognitive tasks provided partial support for its convergent validity. Time monitoring performance in EPELI was associated with monitoring behavior (i.e., fuel checks) in the time-based version of Cruiser, and with performance in the time-based variant of Matching. This indicates that monitoring processes may be similar between the tasks, although the difference in the overall performance variables indicates that other processes are also at play. Monitoring of time as well as environmental cues is a key process of PM that relies on executive functions [25,56,57]. On the other hand, time- and event-based variables in EPELI were not associated with the corresponding measures on conventional PM tasks, indicating that these aspects of the tasks tap on different constructs, or that either of them or both fail to measure real constructs. For example, unlike EPELI, the Cruiser and Matching tasks require simultaneous performance of two tasks (primary task and secondary task) and may not capture all relevant aspects in PM -related processes. More generally, previous research indicates that even structurally similar EF tasks often show poor convergent validity [58]. Reaction time variability in CPT was negatively associated with the EPELI Total Score and both EPELI efficacy scores. This suggests that attentional fluctuations, reflected by the CPT variance, were associated with worse EPELI performance. The two episodic memory tasks showed the most consistent associations with EPELI. This verifies the important role of an episodic memory component in the EPELI game when the participants must remember the list of instructed tasks. These associations are also in line with theoretical accounts postulating a central role of episodic memory in PM [59]. (4) Multiple regression analyses identified age, gender and general intelligence as predictors of EPELI performance. Age-related impairments in PM have been observed especially in conventional PM tasks, but also in ecologically valid contexts [60], concurring with the present findings. Gender effects on PM have not received much attention, but the present gender difference favoring women is in line with other studies that have found gender effects [61]. As one would expect, general cognitive ability showed a positive correlation with EPELI performance, a finding that has been obtained also in other PM studies [62].

### Ecological validity and EPELI

Conventional performance-based tests of PM are considered to have poor ecological validity [7]. We expected EPELI to fare better, but it did not. There are several possible explanations for the lack of associations between EPELI and the questionnaires and diary of everyday PM. First, there is the maximal-typical distinction raised, for example, by Toplak and colleagues [63] in the context of executive function, a cognitive domain that is strongly involved in PM. The conclusion of their review was that performance-based tests of executive functions versus self-reports assess different mental constructs, due to a limited time in the testing which enables maximal effort. More generally, behavior may be biased towards maximal performance when participants know that they are performing an experiment. Thus, EPELI might have elicited a strong cognitive effort that would be less common in daily activities that form the basis for self-assessment of PM or executive function. A second concern pertains not only to EPELI, but to any performance-based PM task, that is, a task utilized in psychological experiments in a controlled fashion. Such tests represent time-constrained short-term tasks where performance is initiated immediately after the instructions. This differs from many everyday PM challenges that also involve self-generated intentions and take place in highly complex, non-controlled environments. The speeded, non-delayed and controlled setup in EPELI and the other PM tasks may also lead to different PM strategy use than everyday situations (e.g., there may be more time and more opportunities for utilizing external cues concerning everyday PM challenges) that can also contribute to discrepancies between objective test results and self-ratings.

The lack of associations between the controlled PM tasks (i.e., both EPELI and the conventional PM tasks) and the questionnaires and diary could also be due to the limitations in the latter measures. Simply based on the lack of correlations between the two, it is not possible to say which one lacks ecological validity, until a robust indicator of actual PM performance in real-world situations has been developed (for an attempt in this direction, see [38]). Sugden and colleagues (pp. 24–25) conclude that self-report “measures are most suitable for the measurement of individuals’ concerns and beliefs about their PM ability and the impact of PM failures on their lives, rather than measures of PM ability itself” [7]. In line with this, they cite research indicating that depression and certain personality features (neuroticism, conscientiousness, self-directedness) are significantly associated with self-reported PM performance but not with objective memory test scores [64–66]. Similar issues have been raised with self-reports of EF as well [64]. The self-reports of PM and EF were associated with depression scores in the present study as well, although this did not account for the lack of associations between EPELI and the PM/EF questionnaires. Such problems pertaining to lack of validity in self-reports could be alleviated by more directly assessing everyday PM lapses, as we aimed to do with the PM diary. Our PM diary was clearly suboptimal as the responses had a very limited sampling range, consisting of a single set of questions during five days. This could be amended with, for example, a longer diary period, or methods such as Ecological Momentary Assessment that probe questions multiple times a day and for longer periods of time [67] or informant ratings. As noted by Sugden et al. [7], informant-ratings tend to show stronger associations with performance-based tests than self-reports. A recent study utilized a device that monitors speech in real time, enabling scoring of PM performance from the recordings [38]. However, that study did not include questionnaires which would have enabled assessing their ecological validity.

Another possible reason why EPELI failed to correlate with questionnaires and diary measuring everyday PM performance in adults could be that the EPELI game is nevertheless too dissimilar to the participants’ everyday life. For example, in this computer-based version of EPELI, activities in the virtual environment are executed with mouse clicks instead of moving the whole body, navigating in the apartment happens by clicking on hotspots, and even though the task itself is open-ended, the set of instructions is fixed. It is possible that such features in the task context lead to different cognitive constructs being utilized. This is emphasized by theories of situated cognition, which imply that cognition is embodied, embedded, enactive, and extended [68]. If cognitive processes are partly constituted by the environment with which the participant interacts with, then differences in context would amount to differences in the cognitive processes [6]. This idea is supported by our recent research indicating that cognitive task performance is an adaptive process [69]. However, based on the verisimilitude ratings, one would expect EPELI to nevertheless show stronger correlations with measures of everyday behaviors than the more artificial conventional PM tasks do.

The present results contradict those of the two previous studies with the child version of EPELI that tested children with or without ADHD [14,36]. In those studies, the EPELI performance was significantly related to parent-rated executive problems and ADHD symptoms. Thus, it does not seem to be the case that EPELI as such lacks ecological validity. There are several possible reasons why the child and adult data differ. First, the child studies utilized informant ratings, and as noted above, these ratings have been shown to be more strongly associated with cognitive performance in experimental tasks [7]. Second, the Seesjärvi et al. studies [14,36] utilized head-mounted displays which could increase the similarity of EPELI to real-life prospective memory and executive behaviors. Third, one could speculate whether children would become more engaged and be less self-conscious about being tested for their memory performance. Hence, their performance in the experimental tasks might be reflecting more closely what they “ordinarily do”. Fourth, children may be less facile in supporting their everyday PM with various strategies or skills than adults do and thus show less task-specificity. Finally, our participants were thoroughly screened for psychiatric symptoms, which was not done in the child studies, one of which also included a clinical sample of ADHD participants. Thus, our sample may have been extraordinarily healthy and thereby showing less variance in the key variables. This was indicated as ceiling effects in some of the performance variables and floor effects in some of the questionnaires that tap on PM and EF problems. However, these floor and ceiling effects were not serious, except for the diary.

### Strengths and weaknesses of the present study

The main strength of our study is its large sample size, which should allow a reliable analysis even for small correlations. Thus, at least when considering this specific setup (i.e., home-based testing with adult sample), the lack of correlations between web-browser version of EPELI and the self-reported measures of everyday PM is likely to be a robust finding, which is also supported by the inverted Bayes Factors that indicated evidence for the null hypothesis. On the other hand, the utilization of remote testing could be considered as a weakness of our study, as it neither enables control of the testing environment (e.g., making sure that the tests are performed without disturbances) nor ascertains that the participants carefully answer each item in the questionnaire. However, previous research indicates that online testing can yield similar results as laboratory testing [41,70] and the present study demonstrates the feasibility of conducting a complex, 3D game-based test like EPELI through the Internet.

### Future directions

The ecological validity of both performance-based PM tasks as well as questionnaires is an open question. Future studies could shed light on this by utilizing methods that probe more directly on everyday performance, such as Ecological Momentary Assessment, wearable devices, or informant ratings. It would also be possible to conduct similar tasks as in EPELI in real apartments, which would enable a more direct test of the ecological validity of EPELI. An experiment of this kind is indeed planned by our group. At the same time, it will be of interest to examine the predictive validity of adult EPELI in different clinical groups vs. healthy controls, as clinically feasible objective measures of complex cognition are mostly lacking. To this end, the present data collection included an adult ADHD sample, which will be reported in future research.

## Conclusion

The present study shows that the adult version of EPELI, a new 3D videogame designed to measure prospective memory and related executive functions through web-based self-assessment, shows excellent internal consistency and is rated as much more life-like than conventional prospective memory tasks. Associations with other cognitive tasks and background variables indicate that it entails an episodic memory and monitoring component, and that EPELI performance is related to age, gender and intelligence. However, adults’ EPELI performance was not associated with questionnaires or diary reports of everyday prospective memory performance. Better sampling of real-life prospective memory performance with methods such as Ecological Momentary Assessment could provide more definite answers on the relevance of EPELI performance and PM questionnaires for actual everyday behaviors. Future research will also show the value of EPELI in clinical contexts.

## Supporting information

S1 Table Reliability in EPELI as a function of number of scenarios from the beginning of the task. (DOCX)

S2 Table Correlations between all the self-report measures. (DOCX)
