# Supplementary material for: Assessment of goal-directed behavior with the 3D videogame EPELI: Psychometric features in a web-based adult sample
Source: PLoS One. 2023 Mar 21;18(3):e0280717. doi: 10.1371/journal.pone.0280717 (PMC10030028; doi:10.1371/journal.pone.0280717)
Supplement: S1 Table — (DOCX) [file pone.0280717.s001.docx]

**Supplementary Table A**

*Reliability in EPELI as a function of number of scenarios from the beginning of the task*

|  |  | **Number of scenarios** | | | | | | | | |
| --- | --- | --- | --- | --- | --- | --- | --- | --- | --- | --- |
| **Measure** |  | 10 | 9 | 8 | 7 | 6 | 5 | 4 | 3 | 2 |
| Total score |  | .88 | .86 | .85 | .83 | .81 | .78 | .75 | .68 | .59 |
| Task efficacy |  | .81 | .79 | .78 | .74 | .70 | .67 | .63 | .55 | .52 |
| Navigation efficacy |  | .81 | .79 | .77 | .73 | .70 | .66 | .63 | .57 | .45 |
| Controller motion |  | .96 | .95 | .95 | .94 | .93 | .92 | .92 | .89 | .86 |
| Actions |  | .95 | .94 | .94 | .93 | .92 | .91 | .89 | .85 | .79 |
