# Supplementary material for: Assessment of goal-directed behavior with the 3D videogame EPELI: Psychometric features in a web-based adult sample
Source: PLoS One. 2023 Mar 21;18(3):e0280717. doi: 10.1371/journal.pone.0280717 (PMC10030028; doi:10.1371/journal.pone.0280717)
Supplement: S2 Table — (DOCX) [file pone.0280717.s002.docx]

**Supplementary Table B**

*Correlations between all the self-report measures*

| **Variable** |  | **ASRS A** |  | **PRMQ P** |  | **PRMQ R** |  | **ADEXI** |  | **ADEXI Inhibition** |  | **ADEXI WM** |  | **MPMI PMA** |  | **MPMI PMSE** |  | **MPMI PMSI** |  |
| --- | --- | --- | --- | --- | --- | --- | --- | --- | --- | --- | --- | --- | --- | --- | --- | --- | --- | --- | --- |
| **ASRS A** | Pearson's r | — |  |  |  |  |  |  |  |  |  |  |  |  |  |  |  |  |  |
|  | BF₁₀ | — |  |  |  |  |  |  |  |  |  |  |  |  |  |  |  |  |  |
| **PRMQ P** | Pearson's r | 0.491 | *** | — |  |  |  |  |  |  |  |  |  |  |  |  |  |  |  |
|  | BF₁₀ | 8.57E+13 |  | — |  |  |  |  |  |  |  |  |  |  |  |  |  |  |  |
| **PRMQ R** | Pearson's r | 0.489 | *** | 0.831 | *** | — |  |  |  |  |  |  |  |  |  |  |  |  |  |
|  | BF₁₀ | 6.14E+13 |  | 5.06E+62 |  | — |  |  |  |  |  |  |  |  |  |  |  |  |  |
| **ADEXI Total** | Pearson's r | 0.558 | *** | 0.647 | *** | 0.588 | *** | — |  |  |  |  |  |  |  |  |  |  |  |
|  | BF₁₀ | 9.31E+15 |  | 5.08E+23 |  | 1.90E+18 |  | — |  |  |  |  |  |  |  |  |  |  |  |
| **ADEXI Inhibition** | Pearson's r | 0.493 | *** | 0.361 | *** | 0.314 | *** | 0.761 | *** | — |  |  |  |  |  |  |  |  |  |
|  | BF₁₀ | 4.03E+11 |  | 202011.7 |  | 4790.169 |  | 2.16E+38 |  | — |  |  |  |  |  |  |  |  |  |
| **ADEXI WM** | Pearson's r | 0.492 | *** | 0.678 | *** | 0.623 | *** | 0.944 | *** | 0.503 | *** | — |  |  |  |  |  |  |  |
|  | BF₁₀ | 3.84E+11 |  | 9.72E+26 |  | 1.96E+21 |  | 5.64E+99 |  | 1.66E+12 |  | — |  |  |  |  |  |  |  |
| **MPMI PMA** | Pearson's r | 0.423 | *** | 0.575 | *** | 0.506 | *** | 0.518 | *** | 0.331 | *** | 0.52 | *** | — |  |  |  |  |  |
|  | BF₁₀ | 7.71E+07 |  | 1.56E+17 |  | 2.26E+12 |  | 9.57E+12 |  | 14593.5 |  | 1.37E+13 |  | — |  |  |  |  |  |
| **MPMI PMSE** | Pearson's r | 0.076 |  | -0.194 |  | -0.167 |  | -0.063 |  | 0.057 |  | -0.113 |  | -0.011 |  | — |  |  |  |
|  | BF₁₀ | 0.157 |  | 4.802 |  | 1.635 |  | 0.131 |  | 0.121 |  | 0.329 |  | 0.087 |  | — |  |  |  |
| **MPMI PMSI** | Pearson's r | 0.047 |  | 0.013 |  | 0.028 |  | 0.147 |  | 0.101 |  | 0.144 |  | 0.227 | * | 0.465 | *** | — |  |
|  | BF₁₀ | 0.108 |  | 0.087 |  | 0.093 |  | 0.824 |  | 0.251 |  | 0.748 |  | 22.159 |  | 9.55E+09 |  | — |  |
| **Diary, average number of PM lapses** | Pearson's r | 0.424 | *** | 0.543 | *** | 0.503 | *** | 0.405 | *** | 0.294 | *** | 0.39 | *** | 0.416 | *** | -0.08 |  | 0.02 |  |
|  | BF₁₀ | 5.35E+07 |  | 2.79E+14 |  | 6.99E+11 |  | 7.53E+06 |  | 889.037 |  | 1.79E+06 |  | 2.29E+07 |  | 0.167 |  | 0.091 |  |
| *  BF₁₀ > 10, ** BF₁₀ > 30, *** BF₁₀ > 100 | | | | | | | | | | | | | | | | | | | |

Note: ASRS A = Adult ADHD Self-Report Scale Part A (range from 0 to 4); PRMQ = The Prospective Retrospective Memory Questionnaire (P = prospective subscale; R = retrospective subscale) (range from 1 to 5); ADEXI = Adult Executive Functioning Inventory (Total score, Inhibition = Inhibition subscale score, WM = Working Memory subscale score) (range from 1 to 5); MPMI = Metacognitive Prospective Memory Inventory (PMA = prospective memory ability; PMSE = use of external aids; PMSI = use of internal aids) (range from 1 to 5). The mean value in the diary reflects the average amount of PM lapses made per day during the five days (theoretical range from 6 to 30 where 6 reflects no PM lapses; see the subsection *Diary questions about everyday PM* for details).
